# Acute Stress Reduces the Social Amplification of Risk Perception

**DOI:** 10.1038/s41598-020-62399-9

**Published:** 2020-05-12

**Authors:** Nathalie F. Popovic, Ulrike U. Bentele, Jens C. Pruessner, Mehdi Moussaïd, Wolfgang Gaissmaier

**Affiliations:** 1 Graduate School of Decision Sciences and Zukunftskolleg, University of Konstanz, Konstanz, Germany; 2 Department of Psychology, University of Konstanz, Konstanz, Germany; 3 Centre for the Advanced Study of Collective Behaviour, University of Konstanz, Konstanz, Germany; 4 Center for Adaptive Rationality, Max Planck Institute for Human Development, Berlin, Germany

**Keywords:** Human behaviour, Endocrinology

## Abstract

Risk perceptions typically underlie a complex social dynamic: Risk-related information is transmitted between individuals, this information influences risk perceptions, and risk perceptions influence which information is transmitted. This can lead to a social amplification of risk. We test how stress, a widespread affective state, influences the social dynamics of risk perception. Participants (*N* = 146) read articles about the controversial antibacterial agent Triclosan and were then asked to inform another person about Triclosan. Before and after reading the articles, participants reported their concern about Triclosan. Stress exposure before the task led to a smaller increase in concern in response to the articles. The stronger the increase in cortisol, the smaller the increase in concern. Furthermore, participants in the stress group transmitted less negative information about Triclosan to others. In contrast, participants’ subjective feelings of stress were associated with higher concern and more alarming risk communication. We conclude that feeling stressed can amplify risk perception, whereas the endocrine stress reaction can attenuate risk perception when information about risk is exchanged in a social context.

## Introduction

Within the context of a growing impact of mass media and social networks, understanding the social dynamics behind the formation of risk perceptions becomes an increasingly important task. People’s perceptions of a potential threat are often not in line with scientific evidence and can be based on misinformation^1,2^. Information about a particular risk received through media sources or social networks is likely to influence these perceptions^3^. When this risk-related information is passed on to other individuals through means of social media or verbal communication, parts of the information typically gets altered or even lost^4^. People tend to convey information that is in line with their initial risk perception, neglecting opposing information. This, in turn, can lead to an amplification of the initial risk perception of the group, even if the original information supported the opposite view; it also fuels polarization between different groups^4^.

Little is known about how acute stress, a widespread affective state, influences the individual and social dynamics of risk perception. Through the activation of the hypothalamic–pituitary–adrenal (HPA) axis and the subsequent release of the stress hormone cortisol^5^, stress is known to influence memory processes^6–8^ and decision making under risk and uncertainty^9,10^. To the best of our knowledge, only one study has looked at the relation between acute stress and risk perception so far^11^. In this study, participants were asked to imagine consequences of different risky situations (e.g., “Ignoring persistent medical problems”) and to indicate their risk perception of each situation. The authors found no direct but an indirect effect of acute stress on risk perception: Participants who were exposed to a stressor prior to the task rated the situations as more stressful and, consequently, also as more risky.

Whereas previous research^11^ investigated individual risk perceptions, we emphasize the importance to understand risk perception as a social, dynamic process^4^. Risk perceptions are rarely formed in isolation from others, but rather they are part of a wider context and communication process. According to the social amplification of risk framework^12^, information about a risk is transmitted from one station to the next in a communication chain. At each station (which can be an individual, media outlet, or an institution), the signal of the risk may be amplified or attenuated. In the current study, we investigated how acute stress influences this amplification or attenuation process. This implies asking whether stress influences how individuals perceive a risk, how they change this risk perception when receiving information about the risk, and what kind of information about the risk they transmit to the next individual. Since we are often under acute stress in our everyday lives and information about a risk itself might already trigger a stress reaction^13^, it seems highly relevant to better understand this relation.

We used a slightly adapted version of a previously developed paradigm for studying the social dynamics of risk perception^4^. In this paradigm, participants are asked to read articles about a controversial chemical substance, the antibacterial agent Triclosan, and subsequently pass on information to another participant informing him or her about Triclosan. Participants report their risk perception of Triclosan before and after reading the articles. Whereas our previous research^4^ studied transmission of risk information in social diffusion chains of ten participants, we explore the effect of stress on the attenuation or amplification process at *one* chain position, that is, of one participant. Each participant received the same risk information and passed on this information by writing a message to another person. Just prior to this task, half of the group was exposed to a group-version of the Trier Social Stress Test^14^ (TSST; involving public speaking and mental arithmetic in front of an audience), while the other half completed a control task. The TSST is a well validated stressor which leads to reliable increases in subjective stress perception, cortisol release, and activation of the sympathetic nervous system^15^. Note that this stress induction was completely unrelated in terms of its content to the communicated risks of Triclosan, which is important, as we are interested in the general effects of the physiological state of being stressed or not on risk perception. To validate the induction of stress, we measured participants’ subjective stress as well as their cortisol and alpha-amylase (a marker of autonomic nervous system activity) levels through saliva samples in twelve- to fifteen-minute intervals throughout the experiment. The study has been reviewed and approved by the Institutional Review Board of the University of Konstanz (IRB 12-2017), and this research was performed in accordance with the relevant guidelines and regulations concerning behavioral experiments with humans.

Our analyses focused on understanding the influence of acute stress on (1) initial risk perception, (2) change in risk perception and the influence of the presented information, and (3) the signal of the transmitted risk information. For the main research questions, we compared the variables of interest (risk perception, change in risk perception and influence of risk information, message signal) among the stress group and control group. To get a better understanding of the mechanisms underlying the effects of stress, we additionally explored associations between hormonal stress responses and the variables of the risk task. Based on previous findings^11^, we also tested for indirect effects of acute stress on risk perception through higher subjective stress ratings. Regarding the effects of stress on change in risk perception, on the influence of risk information and on the transmission of risk information, our analysis remain explorative due to limited research on these topics.

## Results

### Sample characteristics

Equal distribution of potentially confounding variables across the two groups (stress, control) was tested using two-sample t-tests and Mann-Whitney tests for age, body mass index (BMI), self-esteem, perceived stress and time of testing. Chi-square tests were used for gender, smoking, use of contraceptives, menstrual cycle phase, medication intake and group size during testing. None of these variables occurred more frequently in one of the two groups, all *p*s > 0.05 (see Table 1).

**Table 1:** Participant characteristics of the study sample in sum, the stress (*n* = 73) and the control group (*n* = 68).

	Sum	Control Group	Stress Group	*p*^c^
Age [yr]	23.29 (3.94)	23.13 (3.87)	23.43 (4.03)	0.551
Gender (women/men)	68/73	33/35	35/38	0.945
BMI^b^ [kg/m^2^]	22.51 (2.96)	22.32 (3.13)	22.70 (2.80)	0.416
Smoking^b^ (no/yes)	109/31	50/17	59/14	0.378
Medication intake (no/yes)	122/19	61/7	61/12	0.286
Contraceptive use (no/yes/men)	28/40/73	13/20/35	15/20/38	0.956
Menstrual cycle^a,b^ (FP/LP/men)	41/23/73	19/13/35	22/10/38	0.716
Group size^a^ (2/3/4 subjects)	20/52/69	8/26/34	12/26/35	0.727
Time^a^ [hh.mm]	14.07 (01.36)	14.20 (01.26)	13.55 (01.43)	0.379
PSS^b^	25.23 (6.81)	25.50 (7.30)	24.97 (6.35)	0.648
RSES	21.45 (5.37)	21.77 (6.04)	21.16 (4.69)	0.319

*Note*. Mean values (± standard deviations) or absolute frequencies of participant characteristics. BMI = body mass index, FP = Follicular phase, LP = Luteal phase, PSS = Perceived Stress Scale, RSES = Rosenberg Self-Esteem Scale.

^a^at day of testing.

^b^due to missing values analyses depend on a sample of *n* < 141 (BMI: *n* = 140, smoking: *n* = 140, menstrual cycle: *n* = 137, PSS: *n* = 140).

^c^p-values result from two-sample t-tests (for perceived stress), Mann-Whitney tests (for age, BMI, self-esteem, time of testing) and Chi-square tests (for gender, smoking, use of contraceptives, menstrual cycle phase, medication intake and group size during testing).

### Manipulation check

**Figure 1:**
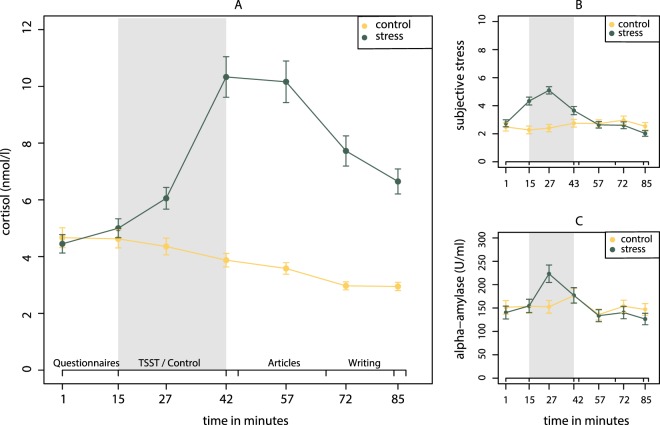
Mean values across participants for the seven time points during the experiment for cortisol levels (**A**), self-reported subjective stress (**B**), and alpha-amylase levels (**C**). Bars denote ± one standard error of the mean.

The stress manipulation successfully increased participants’ cortisol levels. [Fig. 1A, two factor mixed design ANOVA with group (control or stress) as the between subject factor, time (seven levels) as the within subject factor and gender as a covariate: *F*_*stress*_(1, 138) = 48.90, *p* < 0.001; *F*_stress*time_(6, 834) = 54.70, *p* < 0.001]. Initial cortisol levels measured at the beginning of the experiment did not differ between the two groups [*M*_control_ = 4.67 (*SD* = 2.88), *M*_stress_ = 4.45 (*SD* = 2.77), *t*(137.34) = 0.45, *p* = 0.650, *d* = 0.08]. As displayed in Fig. 1C, we found a corresponding activation of the autonomic nervous system in the stress group as indicated by participants’ increased salivary α-amylase levels during the TSST compared to the control task [*M*_control_ = 152.57 (*SD* = 110.61), *M*_stress_ = 223.26 (*SD* = 159.42), two-sample t-test: *t*(128.7) = −3.08, *p* = 0.003, *d* = −0.51].

These findings are in line with participants’ self-reported stress levels: participants in the stress group reported higher values than participants in the control group (two factor mixed design ANOVA with experimental group as the between subject factor, time (seven levels) as the within subject factor and gender as covariate, *F*_*stress*_(1, 138) = 5.64, *p* = 0.019). As can be seen in Fig. 1B, this difference was driven by clearly higher levels of subjective stress during the TSST than during the control task.

We further checked the potential association of group size and gender on these basic variables. Group size did neither have an effect on participants’ hormonal nor on subjective stress responses. There was neither a main effect of gender nor an interaction effect of gender and stress manipulation on cortisol and on α-amylase levels [cortisol: F_gender_(1, 137) = 1.54, p = 0.216; F_stress*gender_(1, 137) = 0.10, p = 0.757; alpha-amylase: F_gender_(1, 137) = 1.14, p = 0.289; F_stress*gender_(1, 137) = 1.18, p = 0.280; see Supplementary Fig. S2]. However, there was a gender effect on subjective stress [*F*_*gender*_(1, 138) = 14.07, *p* < 0.001] with female participants reporting higher subjective stress levels than male participants overall [*M*_female_ = 3.53 (*SD* = 1.94), *M*_male_ = 2.42 (*SD* = 1.65), *t*(132.05) = 3.65, *p* < 0.001, *d* = 0.62; see Supplementary Fig. S2]. Note that the effect of our stress manipulation on subjective stress levels was the same for both female and male participants [F_stress*gender_(1, 137) = 0.01, p = 0.946].

In subsequent analyses, we added gender as a control variable to our models. We also tested for potential interaction effects between gender and stress on all the outcome variables in additional analyses.

### Effects of stress on risk perception and change in risk perception

**Figure 2:**
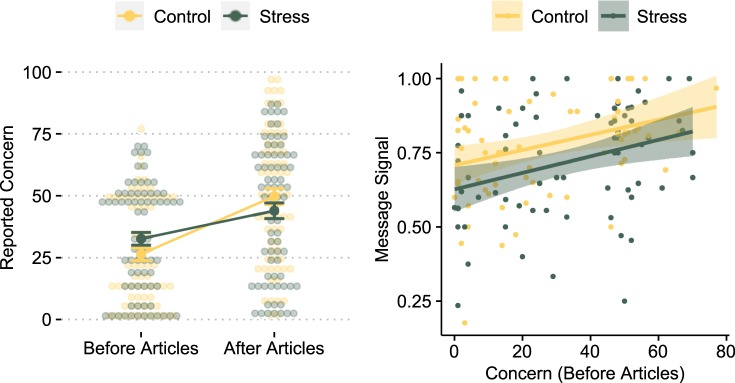
(**A**) Participants‘ reported concern about Triclosan for both experimental groups (control and stress) asked before and after reading the articles about Triclosan. Despite large inter-individual variation, stressed participants changed their reported concern to a substantially lower degree in response to the articles than participants in the control group. Dots represent single individuals, bars denote ±1 standard error of the mean. (**B**) Message signal as a function of experimental group and reported concern about Triclosan before reading the articles. Higher values in message signal indicate a more negative evaluation of Triclosan.

Risk perception was assessed before and after reading the articles. Participants reported how concerned they were about Triclosan on a scale from 0 (not concerned at all) to 100 (extremely concerned). Results are displayed in Fig. 2A. Overall, the majority of participants was initially not very concerned about Triclosan [*M* = 29.62 (*SD* = 21.76), *Md* = 25]. Participants in the stress group were slightly more concerned about Triclosan before reading the articles than participants in the control group [*M*_control_ = 26.41 (*SD* = 21.23), *M*_stress_ = 32.62 (*SD* = 21.96), *t*(138.81) = −1.71, *p* = 0.090, *d* = −0.29]. Interestingly, participants in the stress group changed their reported concern to a substantially smaller degree in response to the articles compared to participants in the control group [change in concern: *M*_control_ = 23.35 (*SD* = 21.89), *M*_stress_ = 11.34 (*SD* = 21.37), *t*(137.75) = 3.29, *p* = _._0.001, *d* = 0.56]. This is also reflected in an interaction effect in a two factor mixed design ANOVA with experimental group (stress or control) as the between subject factor, time point (before articles or after articles) as the within subject factor, and gender as a covariate [*F*_stress*time_point_(1, 139) = 10.86, *p* = 0.001]. Whether the effect of acute stress on change in concern can be (partly) explained by an increase in the stress hormone cortisol is explored further below.

There was no main effect of gender on overall risk perception [*F*_gender_(1, 137) = 1.29, *p* = 0.258], but we did find an effect of gender on change in risk perception: Female participants increased their concern overall to a stronger degree than male participants [change in concern: *M*_male_ = 12.81, *M*_female_ = 21.78, *t*(133.3) = 2.41, *p* = 0.017]. However, our stress manipulation did not effect male and female participants differently: There was no interaction between gender and stress in a three-factor mixed design ANOVA with risk perception as outcome variable, experimental group (stress or control) and gender (male, female) as the between subject factors and time point (before articles or after articles) as the within subject factor [*F*_stress*gender_(1, 137) = 0.25, *p* = 0.622].

### Effects of stress on the influence of risk information

Change in risk perception can be modeled as a combination of one’s initial risk perception, the signal of the message received, and an influence factor that describes the extent to which the received information actually influences one’s risk perception^4^:

1 $${C}_{i}-{C}_{i}^{0}=\alpha (s-{C}_{i}^{0}),$$

where ${C}_{i}^{0}$ is a participants’ initially reported concern divided by 100 to lie between 0 (not concerned at all) and 1 (extremely concerned), ${C}_{i}$ is the reported concern after having received information on the risk divided by 100, $s$ is the signal of the information received, and $\alpha$ is the influence factor with values between 0 (no influence) and 1 (complete adoption). The message signal $s$, meaning the degree to which the information transferred a negative signal about Triclosan, was defined by the number of negative statements relative to the total number of positive and negative statements in the articles or participants’ messages, respectively [s = n^+^/(n^+^ + n^−^)]. The influence factor in previous research^4^ was estimated to take the value of $\alpha =0.45$. Entering the mean values for ${C}_{i}$ and ${C}_{i}^{0}$ for both groups of our experiment separately and the signal of the articles (${s}_{articles}=0.81$) into the formula above, we get a very similar value for the influence factor of the control group (${\alpha }_{control}=0.43$). This value is, however, much lower for the stress group (${\alpha }_{stress}=0.23)$. This suggests that after acute psychosocial stress, people are less influenced in their risk perception by new information.

### Effects of stress on the message signal

The messages that participants wrote to inform another person about Triclosan were on average 214 words long, and length did not differ between the groups [*M*_control_ = 208 (*SD* = 86), *M*_stress_ = 219 (*SD* = 77), *t*(134.5) = −0.78, *p* = 0.439, *d* = −0.13]. Participants’ messages contained a smaller proportion of neutral statements about Triclosan than the original articles, whereas the proportion of positive and negative statements was higher in participants’ messages compared to the articles (see Table 2). In the control group, the proportion of negative statements increased more than the proportion of positive statements. The opposite is the case for participants in the stress group, where the proportion of positive statements increased more than the proportion of negative statements. As a result, the messages of the control group contained a significantly higher proportion of negative statements than messages of the stress group [proportion of negative statements: *M*_control_ = 43% (*SD* = 14%), *M*_stress_ = 37% (*SD* = 15%), *t*(138.93) = 2.74, *p* = 0.007, *d* = 0.46].

**Table 2:** Percentage of neutral, positive and negative statements about Triclosan in the articles, and in messages of participants in the control and stress group.

Valence of Statements	Articles	Control Group (Difference to Articles)	Stress Group (Difference to Articles)
neutral	55%	44% (−11%)	49% (−6%)
positive	9%	13% (+4%)	14% (+5%)
negative	36%	43% (+7%)	37% (+1%)

Replicating previous findings^4^, the message signal *s* was positively correlated with participants’ initially reported concern about Triclosan [*r*(139) = 0.287, *p* < 0.001, see Fig. 2B], indicating that participants were biased by their initial risk perception when transmitting information. To test whether stress leads to an increased bias of transmitting information in line with one’s own risk perception, we ran a linear regression analysis with the message signal (ranging from 0 to 1, with higher values indicating a more negative signal) as dependent variable, and the experimental manipulation (stress or. control), initial concern and an interaction between both variables as independent variables, additionally controlling for gender. Results are reported in Table 2 (Model 1). We did not find an interaction effect between initial concern and our stress manipulation on the message signal, indicating that acute stress does not increase the bias to transmit information corresponding to one’s risk perception. Excluding the interaction term from the model improved model quality (as indicated by a lower AIC) and showed again a negative effect of stress on the message signal (see Table 3, Model 2): Participants’ message signal in the stress group was less negative with respect to Triclosan than the message signal in the control group. This finding is well in line with the finding that participants in the stress group reported a smaller change in their risk perception about Triclosan in response to the articles. Overall, these results suggest that under acute stress, people focus less on negative aspects of risk-related information and get less worried by negative information.

**Table 3:** Regression analyses on the effect of stress manipulation and initial concern on participants’ message signal.

Predictors	Message Signal			
	Model 1		Model 2	
	B (CI)	p	B (CI)	p
(Intercept)	0.730 (0.657–0.802)	**<0.001**	0.728 (0.666–0.790)	**<0.001**
Initial Concern	0.003 (0.001–0.005)	**0.010**	0.003 (0.001–0.004)	**<0.001**
Stress Manipulation	−0.079 (−0.177–0.020)	0.116	−0.075 (−0.133 – −0.016)	**0.012**
Gender	−0.044 (−0.102–0.014)	0.135	−0.044 (−0.101–0.013)	0.133
Stress Manipulaion*Initial Concern	0.000 (−0.003–0.003)	0.920		
Observations	141		141	
R^2^/adj. R^2^	0.138/0.112		0.138/0.119	
F-statistics	5.431***		7.291***	
AIC	−87.512		−89.502	

### Effects of individual stress responses

#### Hormonal stress responses

So far, all of the analyses were concerned with mean differences between the stress and the control group. An important next step is to analyze whether these mean differences are mediated by individual hormonal stress responses. In other words, is the effect of acute stress on changes in risk perception and on the signal of the transmitted message associated with an increased release of the hormone cortisol? To test this, we computed for each participant the area under the curve with respect to increase (AUC_I) of the cortisol measures^16^ and tested its correlation with our variables of interest, change in risk perception and message signal.

**Figure 3:**
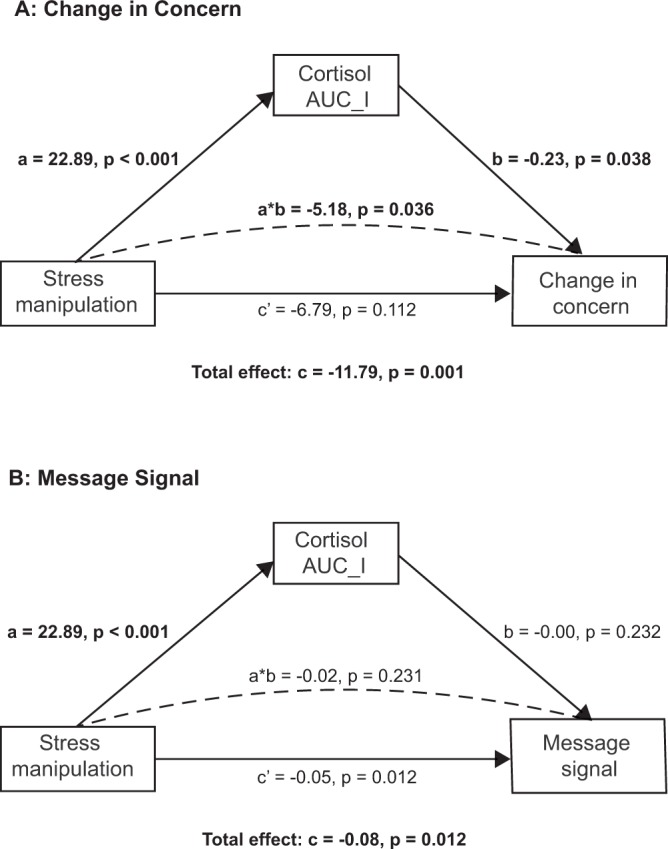
Mediation models with unstandardized coefficients for the indirect effect of the stress manipulation on participants’ change in concern (**A**) and message signal (**B**) through the increase in cortisol.

Participants’ change in risk perception was linked to their hormonal stress response: the higher the cortisol increase over the course of the study, the smaller the increase in risk perception [*r*(140) = −0.298, *p* < 0.001]. To further analyze this effect, we conducted a mediation analysis using quasi-Bayesian Monte Carlo method for variance estimation based on 10,000 simulations. In both, mediator and outcome model, we controlled for gender. Results confirmed that the effect of the stress manipulation on change in concern was mediated by increases in cortisol (a*b = −5.18, *p* = 0.036; see Fig. 3A).

For message signal, however, we did not find a corresponding correlation with cortisol increase [*r*(140) = –0.087, *p* = 0.500]. Additionally, we ran a similar mediation analysis as before but with the outcome model predicting message signal based on the stress manipulation, increase in cortisol (AUC_I_), initial concern, and gender. The effect of the stress manipulation on message signal was not related to an increased release of cortisol (a*b = −0.02, *p* = 0.231, Fig. 3B).

#### Subjective stress reports

Past research has found that exposure to a stressor indirectly influences risk perception through increased ratings of the stressfulness of the risky event^11^. Based on this finding, we tested for the possibility that stress exposure indirectly influenced risk perception in our study through increased ratings of subjective stress.

**Figure 4:**
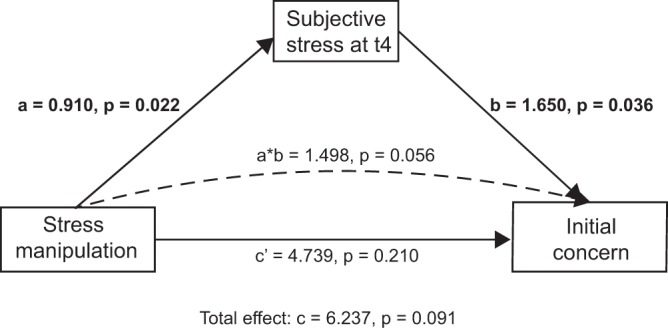
Mediation model for the indirect effect of the stress manipulation on participants’ initial concern about Triclosan through the increase in subjective stress with unstandardized coefficients.

For the analysis of the impact of subjective stress levels, first, we analyzed whether there was a link between how people rated their current feelings of stress and how concerned they were about the risks associated with Triclosan. Reported concern about Triclosan before as well as after the articles significantly correlated with participants’ subjective stress reports at the time of the question, that means at t_4_ and t_7_ [before articles (t_4_): *r*(139) =0.193, *p* = 0.022, after articles (t_7_): *r*(139) = 0.275, *p* = 0.001]. At the same time, subjective stress reports at t_4_ were higher for participants in the stress group compared to participants in the control group [*M*_control_ = 2.75, *M*_stress_ = 3.65, *t*(138.94) = −2.24, *p* = 0.026]. Mediation analyses using quasi-Bayesian Monte Carlo method for variance estimation based on 10,000 simulations also demonstrated a small indirect effect of the stress manipulation on reported concern through subjective stress (a*b = 1.498, *p* = 0.056, controlling for gender in both, mediator and outcome model, Fig. 4). This indicates that, on an individual level, acute feelings of subjective stress are linked to an increased concern about a potential risk. However, *change* in concern in response to information does not seem to be influenced by subjective stress [no correlation between the area under the curve with respect to increase of subjective stress and change in concern: *r(139)* = *−0.03, p* = *0.745*].

Moreover, we found that a higher baseline level in subjective stress (as measured directly at the beginning of the study) was positively related to the proportion of negative statements in participants’ messages [*r*(139) = 0.205, *p* = 0.015].

## Discussion

The presented study investigated the effect of acute stress on the social dynamics of risk perception. More specifically, we tested whether stress influences how people perceive a risk, how they update this risk perception in response to relevant information and what kind of risk information they transmit to another person. The potential risk in our study was the antibacterial agent Triclosan and the information presented to participants were real-world articles with an overall rather concerning message about the substance. Most participants initially reported not to be very concerned about Triclosan. After having read the articles, participants reported increased concern overall. Importantly, we found a substantial difference between participants who were exposed to an acute stressor compared to participants who performed a control task at the beginning of the study: Participants in the stress group were less influenced by the articles and increased their reported concern to a significantly smaller degree. Interestingly, this effect was mediated by increases in the stress hormone cortisol: the stronger the increase in cortisol, the smaller the increase in concern. In line with this finding, participants who were acutely stressed passed on information with a less negative signal about Triclosan than participants who were not acutely stressed, even though this effect was not mediated by increases in cortisol.

These findings suggest that acute stress causing an increase in cortisol renders people to be less responsive to negative risk information. Studies from different domains have shown that acute stress and an elevated cortisol level can lead to a subsequent avoidance of negative information. It has previously been shown that participants who completed a stressful task were subsequently faster in shifting attention away from negative words compared to positive and neutral words in a spatial cueing task^17^. Similarly, acute stress and elevated cortisol levels have been found to reduce selective attention to threat in an emotional stroop task^18,19^. Research on the effects of acute stress on reinforcement learning have shown that after acute stress people show enhanced learning from positive feedback^20^ but impaired learning from negative feedback^21^. There is even evidence that an elevated cortisol level reduces phobic fear^22^ and activity of the amygdala, a brain region known to play a key role in processing of emotions such as fear and anxiety^23^. Whereas the immediate stress response activates the amygdala to increase vigilance^24^, cortisol then seems to reduce this vigilant and anxious state again by desensitizing the amygdala^23^. These findings indicate that adaptive processes are at play after acute stress to regulate emotions and restore homeostasis and that cortisol plays a crucial role in mediating these effects, and terminating the stress response. Our study suggests that a similar process might be involved when people are faced with risk-related information while experiencing acute stress combined with cortisol increases. Neglecting negative information about a potential risk could be a natural defense mechanism to reduce anxiety after an acute threat.

Note that in our study, the risk information presented to participants was completely unrelated to the social threat encountered by the stress group. This may be a crucial point. When information is relevant to the stress context, in contrast, attention towards negative information increases^19^. In a case where the risk itself triggers the stress response (such as, for example, the information about the outbreak of an epidemic), people might become more vigilant to the negative aspect of risk information and become more concerned by the risk information.

The articles in our study had an overall negative signal about Triclosan. We chose those articles because they represent a naturalistic sample of information about a risk (namely real-world online articles found when searching for “Triclosan”) and because they were used in previous research^4^. A remaining question is how stress influences the social dynamics of risk perception in response to information with an overall neutral or positive signal. If people indeed attend selectively to positive and negative information in order to regulate emotions after acute stress, participants who have been acutely stressed should be influenced more strongly by risk information if this information is reassuring.

Our findings imply that there is a substantial difference in how people deal with risk information just after they have been exposed to a stressor that is not present anymore but still has an influence on HPA axis activation and circulating cortisol levels, and in how people deal with risk information when they currently feel subjectively stressed. The more people reported to feel stressed at the moment of the risk perception question, the more they also reported to be concerned about Triclosan. Similar to related research^11^, we did not find a direct effect of acute stress on initial risk perception (reported by participants before reading articles about the risk) but an *indirect* effect through elevated subjective stress. In this research^11^, participants were presented with different hypothetical situations and were asked as how risky they rated each situation. Subjective stress was measured by asking participants how stressful they would rate the presented situation. In their study, the subjective stress measure was, hence, directly associated with the potential risk. Our results show that even when the subjective stress measure is completely unrelated to the risk (the fourth stress measurement happened just shortly before the first risk perception question about Triclosan and before participants have gotten any information about the chemical), it still influences how people perceive the risk. We also found that participants with higher baseline levels of subjective stress passed on a higher proportion of negative information in their messages. How do these findings fit with the findings from the group comparison and individual cortisol analyses? Note that during the TSST, participants reported significantly increased levels of subjective stress but these subjective stress levels quickly returned to baseline after conclusion of the TSST (see Fig. 1B). Cortisol had its peak right after the TSST and remained above the level of the control group throughout the experiment (see Fig. 1A). Such a lagged association between psychological and endocrine stress responses is also reported in other studies^25,26^. Our results show that the physiological reaction to a stressor can still have an influence on behavior although people overall do not feel subjectively stressed anymore.

What do our findings reveal about the influence of stress on the social amplification or attenuation process of risk perception, given that people tend to transmit risk information that is consistent with their preconceptions^4^? Our results replicated this finding: Participants’ message signal was positively associated with their initially reported concern, irrespective of the stress manipulation. Although stress in our study did not influence this bias to transmit information consistent with one’s beliefs, participants in the stress group were less influenced by the received information and transmitted an overall less negative message signal to others. This suggests that acute stress can make the communication about a risk less alarming and hence reduce social amplification of risk. At the same time, subjective stress seems to make a social amplification of the risk signal more likely. Both an amplification and attenuation of the risk can be harmful: Underestimation of a risk can increase incautious actions such as risky driving or practicing unsafe sex. Overestimation of a risk can lead to unnecessary anxiety and dangerous behavior, such as not getting vaccinated. Further research to better understand the differential effects of stress on the social dynamics of risk perception hence seem relevant not only from an individual but also from a policy perspective.

## Methods

### Participants

We recruited 146 young healthy subjects through electronic and paper advertisements at the University of Konstanz. Participants either received 15€ or course credit as a compensation. Four participants were excluded because of very high baseline cortisol levels (larger than 3 standard deviations from the mean) and one because she showed a strong increase of cortisol in the control group with a cortisol level larger than 3 standard deviations from the mean at the third measurement (see Supplementary Fig. S1 for individual cortisol levels over the course of the experiment for both groups). The remaining 141 participants were included in our final analysis [73 male, mean age = 23.29 (*SD* = 3.94)].

### Design and procedure

**Figure 5:**
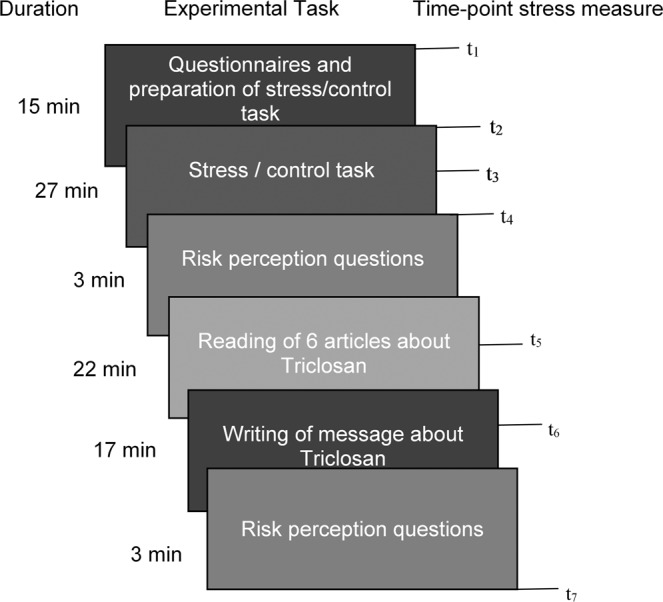
Schematic of experimental procedure. Stress measures included saliva samples and subjective stress reports.

After participants were informed about the procedure of the study and gave their informed consent, they filled out a demographic questionnaire, the Rosenberg Self-Esteem Scale^27^, and the Perceived Stress Scale^28^. These measures served to control for potentially confounding factors. (Note that all analyses presented in the Results section were additionally performed controlling for perceived stress and self-esteem. Neither scores of the Perceived Stress Scale nor scores of the Rosenberg Self-Esteem Scale were found to influence stress reactions and behavior in the risk perception task. They are, hence, not included in the results reported here.) Participants were then randomly assigned to receive either the stress induction or the control task in another room, which is described in detail below. Finally, participants went into a third room in which they performed the main task concerned with risk perception, which is also described in detail below. See Figure  5 for a schematic of the experimental procedure.

#### Stress induction and control task

We conducted a slightly modified version of the Trier Social Stress Test (TSST) for groups^14^. During a 5-minute preparation period in a first room, participants had to first identify a job they would want to apply for, and then identify arguments of why they were a good fit for this position. At the end of the preparation period, participants were invited to enter a test room, and asked to perform a video-taped public speaking task and a mental arithmetic task in front of two confederate interviewers (one male and one female both wearing white laboratory coats). In the public speaking portion, participants had three minutes to deliver an oral presentation of why they thought they were the right candidates for the hypothetical job. Up to four participants were scheduled to participate in the TSST for groups, thus the public speaking went on for a total of twelve minutes. The order among the four participants was randomized, and participants were prevented from seeing each other by poster boards set up between them. All participants could however see the interviewers, who were instructed to refrain from any emotional facial expression and to prompt participants to continue speaking if they stopped before the end of the three minutes. After each participant had finished their speech, they continued with a 3-minute arithmetic task each. In this portion of the test, participants were asked to count down from a 4-digit number in steps of 17 and were told to start over if a mistake was made. The order of the arithmetic task was randomized across participants. Experiments were scheduled in groups of four, but due to late cancellations or no-shows sessions took place in groups of four in 37.5% of cases, in groups of three in 37.5% of cases, and with two participants in 25% of cases. To adjust the time of overall stress exposure, in the instances of three or two participant sessions the individual speaking and math time was adjusted to result in overall identical length of the TSST.

In the control group, participants were asked to identify a job they would want to apply for, and prepare arguments for their choice during the preparation time, and to write down those facts on a piece of paper in the testing room with no interviewers present, but with otherwise identical configuration (poster boards, number of people etc.), as well as to (silently) count down from a 4-digit number in steps of 1. They were explicitly told that their notes are solely for themselves and would not be used by the experimenter. The control task was, hence, similar to the task in the treatment condition with respect to paradigm and procedure, but lacked social evaluation, social interaction, and social threat.

#### Main task on the social dynamics of risk perception

The main task initially assessed prior knowledge about the chemical substance of interest, Triclosan, with the following question: “Have you heard about Triclosan before?”, “Do you know how you could be exposed to Triclosan in your daily life? If yes, please specify.” Participants were then asked to report their risk perception of chemical substances in general and Triclosan specifically. Subsequently, participants read six articles stating different views about Triclosan. The articles were already used previously^4^ and represent a selection from the first page of google search results on “Triclosan”. Sources of the articles were Wikipedia, Focus Online (a German online news magazine), Greenpeace, the German Environment Agency (“Umweltbundesamt”), The Cosmetic, Toiletry and Perfumery Association (CTPA), and a report of Environmental Defense (a Canadian environmental organization). All articles were presented in a random order and each one was displayed for three minutes to the participants. After having read the articles, each participant had 17 minutes to write a message to another participant informing him or her about Triclosan. We told participants that their messages would be shown to another participant later on. At the end of the study, participants were asked once again to report their concern about chemical substances in general and about Triclosan.

### Measures

#### Stress measures

Cortisol and salivary alpha-amylase (sAA) were analyzed from saliva samples (Sarstedt AG & Co, Nümbrecht, Germany). Cortisol levels (nmol/l) were measured using a time-resolved fluorescence immunoassay^29^. Salivary alpha-amylase (U/ml) levels were determined using the enzyme kinetic method^30^. Participants’ subjective stress levels were measured on a visual analogue scale ranging from 0 to 10. All measures were anchored to 7 time-points, in twelve- to fifteen-minute intervals, throughout the experiment from 0’ to 90’ minutes. Missing values of a single time-point were replaced with the mean value of the measurement between the time-point before and after the time-point of the missing value. If the missing value occurred at the overall peak of the measurement, it was replaced with the group mean of the corresponding experimental group. In total, there were 7 values of six participants missing and replaced.

#### Risk perception

To measure participants’ risk perception, we asked how concerned they feel about chemicals in daily products in general and about Triclosan specifically. Concern as a measure of affective risk perception has been found to be strongly linked to intentions for risk reducing behavior such as getting vaccinated^31^. Answers were given on a visual analogue scale ranging from 0 (not concerned at all) to 100 (extremely concerned). As the risk information provided to participants was specifically about Triclosan, we focused in the analysis on initial concern and change in concern about Triclosan and not on reported concern about chemicals in general. Changes in risk perception were calculated by subtracting concern reported before the articles from concern reported after the articles. Note that although the majority of participants has not heard about Triclosan before the experiment (138 out of 146 participants), we still think that their reported concern about Triclosan before having read the articles is a valid measure of their initial risk perception. First of all, participants were told that Triclosan is a chemical substance used in daily products before being asked about their risk perception of Triclosan. Second of all, initially reported concern about Triclosan and reported concern about chemical substances was significantly correlated (*r*(144) = 0.574, *p* < 0.001). We find very similar patterns on the effect of stress on risk perception when looking at participants’ reported concern about chemicals in general (see Supplementary Fig. S3).

#### Message signal

Participants’ written messages were coded with respect to the signal about Triclosan they conveyed. Three independent coders labeled each sentence in the messages of participants as either positive, negative or neutral with respect to Triclosan. Sentences explicitly stating benefits of Triclosan or suggesting that the use of Triclosan is safe or under control were coded as positive, sentences expressing real or suspected harms of the chemical were labeled as negative. Sentences that neither expressed a positive or negative statement about Triclosan, or that expressed both simultaneously, were coded as neutral. Inter-coder reliability was high (*α* = 0.878^32^). The final amount of positive, negative and neutral statements per participant was determined by calculating the mean response of the three coders. The message signal, meaning the degree to which the text transferred a negative signal about Triclosan, was defined by the number of negative statements relative to the total number of positive and negative statements [s = n^+^/(n^+^ + n^−^)], in accordance with previous research^4^.

## Supplementary information

Supplementary Information.

## Data Availability

The datasets generated and analyzed during the current study are available from the corresponding author on reasonable request.
