# Supplementary material for: Acute Stress Reduces the Social Amplification of Risk Perception
Source: Sci Rep. 2020 May 12;10:7845. doi: 10.1038/s41598-020-62399-9 (PMC7217964; doi:10.1038/s41598-020-62399-9)

Supplementary Information

Acute Stress Reduces the Social Amplification of Risk Perception

Nathalie F. Popovic[a], Ulrike U. Bentele[b], Jens C. Pruessner[b,c], Mehdi Moussaïd[d], Wolfgang Gaissmaier[b,c]

[a]Graduate School of Decision Sciences and Zukunftskolleg, University of Konstanz, Konstanz, Germany

[b]Department of Psychology, University of Konstanz, Konstanz, Germany

[c]Centre for the Advanced Study of Collective Behaviour, University of Konstanz, Konstanz, Germany

[d]Center for Adaptive Rationality, Max Planck Institute for Human Development, Berlin, Germany

Figure S1: Individual cortisol levels over the course of the experiment in the control and stress group. Participants with a baseline cortisol level larger than 3 SD plus the mean were excluded from the analysis as well as one participant who showed a strong increase in cortisol in the control group with a cortisol level larger than 3 SD plus the mean at minute 27 (dashed lines).

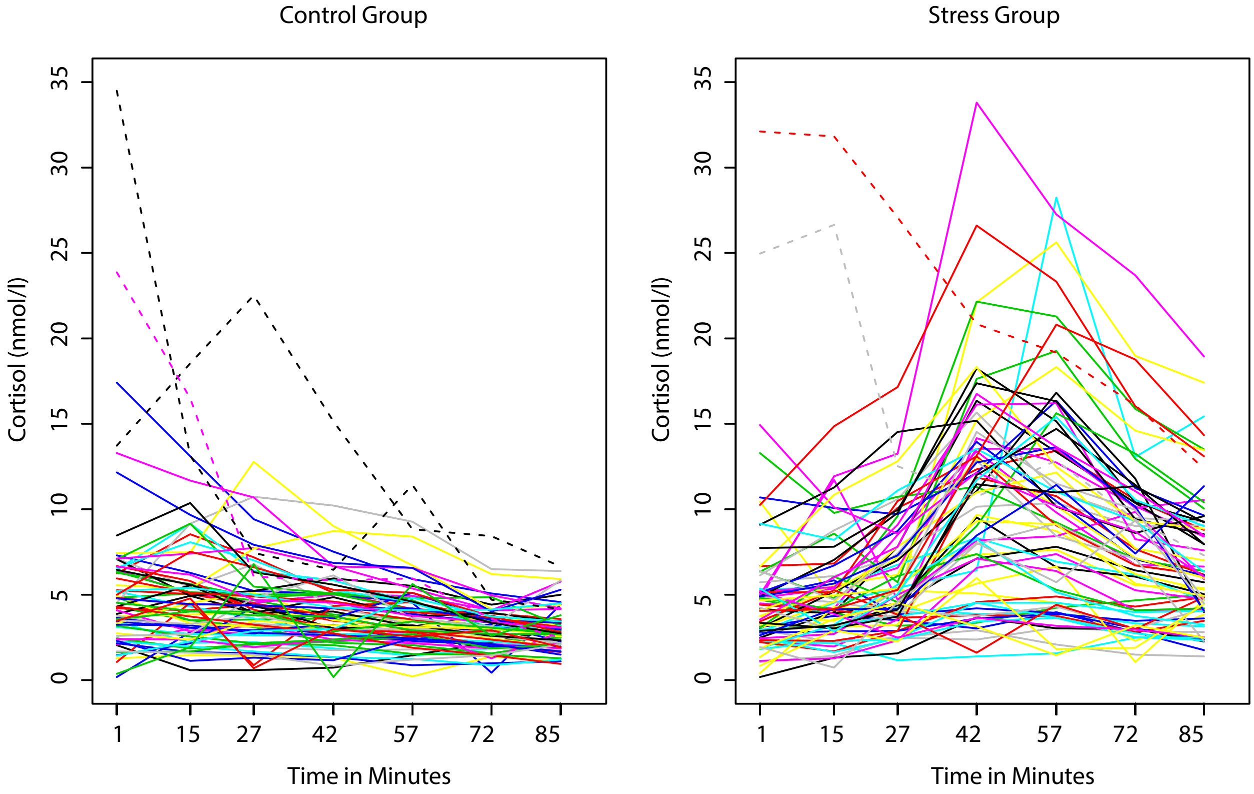

Figure S2: Mean values across female and male participants for the seven time points during the experiment for self-reported subjective stress (A), cortisol levels (B), and alpha-amylase levels (C). Bars denote ± one standard error of the mean.

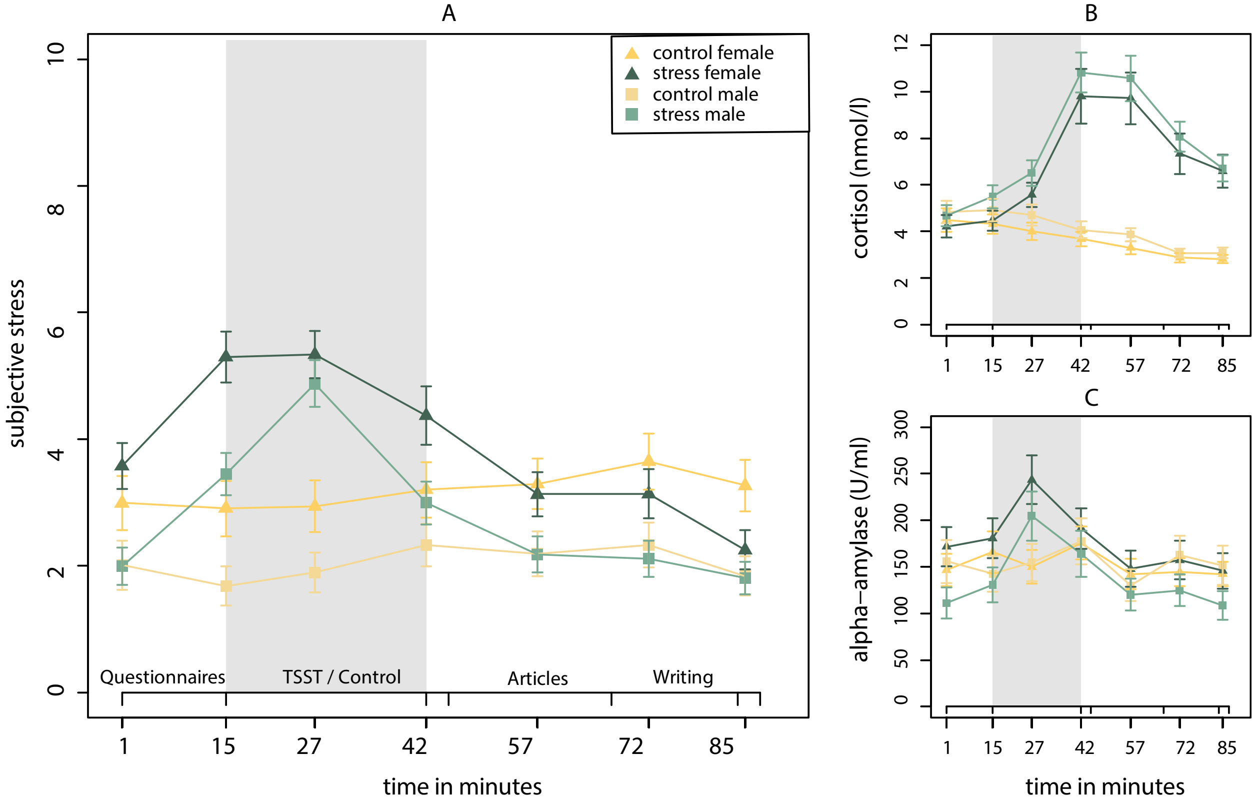

Figure S3: Reported concern about chemicals in daily products in general for both experimental groups (control and stress) asked before and after reading the articles. Similar to findings on reported concern about Triclosan, initially reported concern about chemicals in general did not differ between stress and control group [$M_{control} = 43.87$ ($SD = 27.29$), $M_{stress} = 44.78$ ($SD = 23.48$), $t(132.6) = –0.21$, $p = .832$, $d = 0.04$]. After reading the articles about Triclosan, concern about chemicals in general increases slightly in the control group but not in the stress group [change in concern: $M_{control} = 4.60$ ($SD = 18.44$), $M_{stress} = –0.10$ ($SD = 18.08$), $t(137.86) = 1.53$, $p = .130$, $d = 0.26$]. Dots represent single individuals, bars denote ± 1 standard error of the mean.

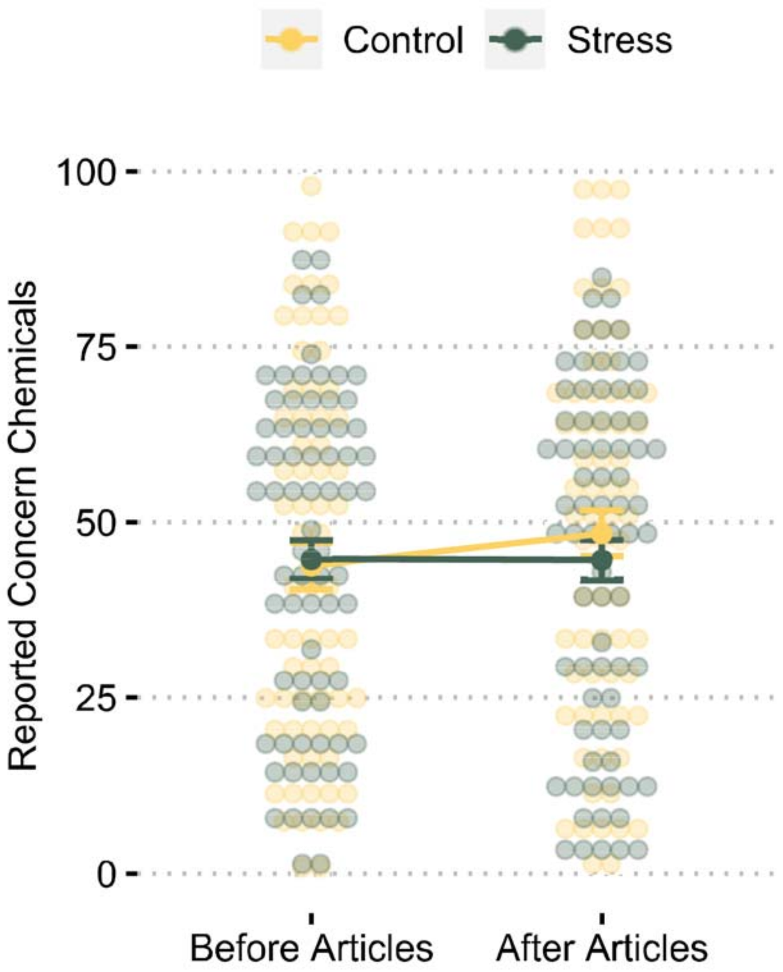

Supplement: Supplementary file 1 — Supplementary Information. [file 41598_2020_62399_MOESM1_ESM.pdf]
